# Ultrasound biosafety: Knowledge and opinions of health practitioners who perform obstetric scans in South Africa

**DOI:** 10.4102/hsag.v24i0.1028

**Published:** 2019-10-17

**Authors:** Salome E. Mashiane, Barbara van Dyk, Yasmin Casmod

**Affiliations:** 1 Department of Medical Imaging and Radiation Sciences, Faculty of Health Sciences, University of Johannesburg, Johannesburg, South Africa

**Keywords:** Obstetric Ultrasound, Ultrasound Bio-Effects, Safety Indices and Principles, Acoustic Output, Mechanical Index, Thermal Index, Teratogenic, ALARA

## Abstract

**Background:**

Diagnostic ultrasound is generally considered as a safe test in pregnancy. To date there is no evidence that ultrasound has caused harm to the developing foetus. However, with the number of obstetric scans on the rise and the steep increase in acoustic output achieved by modern machines, the lack of evidence of absolute safety remains a concern. Acoustic output is under the direct control of the operator and is therefore the operator’s responsibility to keep the intensity as low as reasonably achievable. A situation analysis in the South African context was deemed necessary to determine end user knowledge and opinions on safe antenatal ultrasound practice.

**Aim:**

The aim of this quantitative descriptive, cross-sectional study was to evaluate the knowledge and practice of health practitioners who perform antenatal scans regarding safety aspects of diagnostic ultrasound.

**Setting:**

A self-administered questionnaire was distributed at two national congresses, hosted by the South African Society of Ultrasound and Obstetrics (SASUOG) and South African Society of Obstetricians (SASOG) committees.

**Method:**

Quota non-probability sampling allowed for the identification of professional categories capable of providing information relevant to the study objectives. The sample represented a population with experience in obstetric ultrasound.

**Results:**

Compared to international studies, South African end users demonstrated better knowledge of safety indices than their international counterparts. It is, however, discouraging that end users still demonstrate insufficient knowledge regarding factors contributing to adverse biological effects.

**Conclusion:**

With room for improvement, an effort should be made to comply with international standards through increased training efforts and raising awareness.

## Introduction

The clinical benefits of obstetric ultrasound are well known and it is sometimes viewed as an extension of the human hand in pregnancy management (Joy, Cook & Love 2006:223). Diagnostic ultrasound has gained reputation as a safe test; however, in spite of its apparent safety, ultrasound energy has the potential to cause harm (Sheiner, Freeman & Abramowicz 2007:319) and should, therefore, be used by adequately trained personnel only when medically indicated (BMUS 2009; EFSUMB 2006). Although no independent longitudinal study on human subjects has confirmed long-term adverse effects, ultrasound bio-effects have been observed in animal studies when similar acoustic outputs were employed (Akhtar et al. 2011:981; Sheiner et al. 2007:319). Studies have reported that ultrasound exposure may be associated with adverse outcomes such as growth retardation, delayed speech development, dyslexia and non-right handedness (Marinac-Dabic, Krulewitch & Moore 2002:19; Sheiner, Freeman & Abramowicz 2005:1665). Ironically, the assumption that ultrasound exposure is safe for a foetus is not based on safety data, but rather on the lack of evidence of harm (Bagley, Thomas & DiGiancinto 2011:252). As the preferred modality for foetal monitoring, the assurance of absolute safety thus remains a concern (Piscaglia et al. 2009:6).

The cause for concern arises from the eightfold increase in the maximum allowed acoustic output permitted by the Food and Drug Administration (FDA) for obstetric ultrasound since 1992 (Abramowicz et al. 2008:542; Nelson et al. 2009:140). However, most epidemiological studies on bio-effects have been based on data predating 1992 when exposures to the foetus were much lower than what is the current practice (Abramowicz et al. 2008:542; Nelson et al. 2009:140; Salvessen et al. 2011:625).

Various international professional bodies and ultrasound committees advocate for the use of obstetric ultrasound for medical reasons only (BMUS 2009; EFSUMB 2006). A number of studies in different parts of the world have shown that there is poor knowledge regarding the safe use of ultrasound among end users (Akhtar et al. 2011:981–985; Necas 2010:28–32; Piscaglia et al. 2009:6–11; Sheiner & Abramowicz 2008:499–501); however, to date, no situation analysis has been conducted in the South African context. For the purpose of this article, the term ‘end user’ will be used to denote any health practitioner who utilises obstetric ultrasound (obstetricians and gynaecologists, midwives and sonographers). The aim of the article is thus to explore and describe the knowledge and opinions of healthcare practitioners who perform antenatal scans with regard to the safety aspects of diagnostic ultrasound, which prompted the researcher to investigate the biosafety issues among the end users.

## Theory

Two major biological effects are considered in safety studies, namely, thermal and non-thermal or mechanical effects.

### Thermal bio-effects

As sound travels through tissue, the intensity of the beam is reduced through processes such as reflection, scattering, refraction, absorption and wave front divergence (Duck 2012:8). The consequential loss of energy leads to the rise of temperature in tissues, which is greatest with higher frequencies and higher acoustic power outputs (Duck 2012:8). Thermal bio-effects thus refer to biological changes associated with a rise in temperature in the insonated tissue.

Thermally induced teratogenesis has been reported in many animal and controlled human studies (Abramowicz et al. 2008:550; Church & Barnett 2012:24–25). Foetal bones absorb energy more strongly than the surrounding soft tissue, with a 30-fold increase in the absorption coefficient as foetal bones mature (Duck 2012:14). As the developing foetal brain is within the direct ultrasound beam and in close proximity to the skull (Starrit & Duck 2011:142), the central nervous system is most vulnerable to heat damage, resulting in abnormalities such as encephalocoeles and microphthalmia (Abramowicz et al. 2008:542; Church & Barnett 2012:53).

B-Mode, M-Mode and three-dimensional (3D) ultrasound imaging are unlikely to give rise to thermal injury because the energy is transmitted in short pulses (Joy et al. 2006:223). Conversely, spectral Doppler produces a fixed ultrasound beam which can cause a significant rise in tissue temperature within a relatively short time during flow studies of the maternal or foetal circulations (Maeda & Kurjak 2012:314–315, 2014:182–183). Adverse effects from Doppler ultrasound are most likely to occur in early gestation when cell division is most rapid and foetal blood flow is less well developed and less likely to dissipate heat effectively (Joy et al. 2006:223). However, caution should be exercised in advanced gestation where there is more bone mineralisation and sound reflection at bone and soft tissue interfaces (Salvessen et al. 2011:625). As it is uncertain whether ultrasound-induced tissue heating is adequate to create a hazard in humans, it is imperative that operators apply safety principles while scanning (Duck 2012:16).

### Mechanical bio-effects (non-thermal bio-effects)

Mechanical bio-effects are biological changes that take place when a gas bubble in a liquid experiences the variations in pressure of an acoustic wave. Gas bubbles resonate by expanding in the rarefaction half of the cycle and contracting in the compression half cycle of the wave (Duck 2012:21). As the sound intensity increases, resonance increases exponentially. Once the gas bubble reaches a critical size, it begins to vibrate and disintegrate into smaller bubbles, causing the release of high temperatures, pressure and free radicals, changes in ion transportation and sonoluminescence (emission of light) (Joy et al. 2006:224). This behaviour is termed acoustic cavitation (Duck 2012:14) and ultimately leads to inertial cavitation injury (Joy et al. 2006:224). Mechanical effects are, however, unlikely to occur in obstetric ultrasound because of the absence of gas in the foetal body as well as the fact that the foetus is surrounded by fluid; thus, the mechanical index has less relevance in obstetric scanning (Bly & Van den Hof 2005:574).

The American Institute of Ultrasound in Medicine (AIUM) introduced the Output Display Standard (ODS) to serve as an onscreen warning system for potential thermal or mechanical harm (Bagley et al. 2011:253). The thermal index (TI) provides an estimate of the maximum temperature rise that could occur in tissue during an ultrasound examination and is determined by the ratio of the total acoustic power – the acoustic power required to raise the tissue temperature by 1 °C. This implies that for a TI of 2, the temperature rise is 2 °C, while the actual tissue temperature is 39 °C. Acoustic power thus forms the basis of the thermal index (Abramowicz et al. 2008:543; Bly & Van den Hof 2005:573; Maeda & Kurjak 2012:314). As a predictor of inertial cavitation, the mechanical index (MI) is displayed as a safety index on modern ultrasound scanners and allows the operator to manage acoustic exposure in such a way that the risk of cavitation effects is minimised (Starrit & Duck 2011:56). Although TI and MI may not be perfect indicators for actual thermal and non-thermal risks, they are regarded as the most sensible method of risk estimation (Sheiner et al. 2007:315). Because acoustic output is under the direct control of the operator, it is the operator’s responsibility to keep the intensity as low as reasonably achievable (ALARA) (Sheiner et al. 2007:322) and to minimise the risk to the foetus by keeping scan times as short as possible, avoiding the unnecessary use of Doppler scanning modes, especially in the first trimester, and advocating for the use of ultrasound for medical purposes only (Bly & Van den Hof 2005:572).

## Design and method

A quantitative descriptive cross-sectional survey was conducted at two national congresses hosted by the South African Society of Ultrasound in Obstetrics and Gynaecology (SASUOG) and the South African Society of Obstetricians and Gynaecologists (SASOG). Both professional organisations represent the target population of sonographers, midwives, general practitioners (GPs), obstetricians and maternal-foetal medicine specialists who practise obstetric ultrasound in the public and private health sectors.

A quota non-probability sampling method was employed by identifying professional categories capable of providing relevant information. Although some professional categories may have been over- or under-represented, the main intention was not to link the overall result to how each professional category responded, but to generalise findings to the entire population, thereby making the potential for bias irrelevant.

The adapted questionnaire previously employed in an American study (Sheiner et al. 2007:324) (Table 1) contained 30 items, of which 15 of the original items addressing the knowledge and opinions of health practitioners are presented and discussed in this article. The questionnaire was divided into categories ascertaining the general demographics, opinions on safe practice and knowledge of bio-effects, safety indices and safety statements. End users were surveyed about the frequency of scans deemed appropriate for low-risk pregnancies, safety, risk and limitations of B-mode and Doppler ultrasound applications during pregnancy. Participants’ familiarity with the safety indices and the ALARA principle were also investigated.

**TABLE 1:** Biographical data.

Variables	*N* = 121	%
**Gender**		
Male	53	44
Female	68	56
**Profession**		
Sonographer	23	19
General practitioner/Physician	9	7
Obstetrician and gynaecologist	86	71
Maternal-foetal medicine specialist	3	3
**Place of work**		
Government institution	56	46
Private sector	62	51
Other	3	3
**Years of experience in ultrasound**		
Less than 1 year	2	2
1–2 years	9	7
3–5 years	17	14
6–10 years	29	24
11–15 years	22	18
16–20 years	24	20
More than 20 years	23	19
**Average number of scans performed daily**		
0–2 scans	10	8
3–5 scans	24	20
6–10 scans	30	25
11–15 scans	35	29
More than 15 scans	22	18

*Source*: Sheiner, E., Shoham, V. & Abramowicz, J.S., 2007, ‘What do clinical users know regarding safety of ultrasound during pregnancy?’, *Journal of Ultrasound in Medicine* 26, 319–325. https://doi.org/10.7863/jum.2007.26.3.319

Permission for data collection was granted by the university ethics committees following a peer review process as well as the executive committees. Participation was voluntary and the language used in the questionnaire and information leaflet was aimed at the intellectual level of the respondents, that is, health professionals. By completing the questionnaire and placing it anonymously in a sealed box, the respondents consented to the dissemination of information by implication. Confidentiality and privacy were ensured through anonymous participation.

Data were captured and analysed using the IBM SPSS version 23 software package. Computed descriptive statistics were employed to present data as frequencies and percentages. Inferential statistical methods were employed to compare multiple variables in −2 × 2 tables, using the Pearson’s chi-squared test to determine statistical significance with *p* < 0.05.

### Ethical consideration

The faculty of Academic Ethics Committee confirm that the research complies with the approved ethical standards of the Faculty of Health Science, University of Johannesburg (Ethical clearance number AEC51-01-2013)

## Results

A total of 515 questionnaires were distributed at two national congresses. Although 159 questionnaires were returned, 38 had to be disqualified because of missing data. Statistical analysis was thus performed on 121 complete data sets.

Obstetricians accounted for the largest professional component (71%), followed by sonographers (19%), GPs (7.4%) and maternal-foetal medicine specialists (2.5%). The majority (71%) had more than 6 years of experience in ultrasound; 46% were employed in the government sector and 51% practised in the private sector; 72% performed more than six scans daily. The sample in general represented a population with experience in obstetric ultrasound.

Cross-tabulations were employed to interrogate the association between demographic variables (i.e. professional categories and experience in ultrasound) and opinions regarding safe scanning practices.

Only 29 respondents (24%) indicated that the use of Doppler test in the third trimester should be limited and only used when medically indicated. The results yielded a statistical significant difference (*p* = 0.017) between the professionals’ opinions.

The majority of sonographers (91%) were familiar with the ALARA term, while 67% of the GPs, 33% of the obstetricians and 33% of the materno-foetal medicine specialists knew the correct meaning of the acronym. A significant statistical difference (*p* = 0.003) was recorded between professional categories and knowledge of the ALARA principle as applicable to ultrasound.

Tests for association revealed that the trend for selecting the correct meaning of ALARA increased as the number of scans performed daily increased, indicating that end users who had performed more scans daily were more aware of the ALARA principle (*p* = 0.02).

The Pearson’s chi-squared test for independence indicates a statistically significant association (*p* = 0.019) between experience in ultrasound and opinions regarding the limitation on the number of scans appropriate in low-risk pregnancies. This indicates that with more experience more caution is exerted regarding the frequency of scans in low-risk pregnancies.

## Discussion

### Knowledge of and opinions on ultrasound potential for bio-effects

Although it has not been proven that obstetric ultrasound has adverse biological effects, the absence of proof of absolute safety dictates mindfulness of the potential for unidentified risks. It is thus vital to maintain a wide margin of safety to account for the uncertainty about thresholds for damage to the embryo and foetus to maintain a safe record for all ultrasound examinations (Barnett 2002:387; Bly & Van den Hof 2005:537). Subtle effects, such as non-right-handedness in boys, can therefore not completely be dismissed (Salvesen 2012:129). Although low, 35.5% of end users in our study acknowledged the potential for adverse effects to the foetus during ultrasound scans (Table 2) as opposed to 11.5% in the American study (Sheiner et al. 2007:322) and 25% in the Pakistani study (Akhtar et al. 2011:983). Some South African participants who selected the ‘no’ option qualified their choice by expressing the possibility of adverse effects if safety precautions are not adhered to. The purpose of the ODS was to provide ultrasound users with a tool to operate equipment safely at higher output levels. Although the ODS did not specify the upper output limits, a TI of 1.5 is generally regarded as the universal threshold (Abramowicz et al. 2008:550; Church & Barnett 2012:24–25; Duck 2008:1339). It is therefore of concern that only 44% of respondents in our study were aware of the potential teratogenic effects of temperature in the first trimester (Table 3), although this percentage is higher compared to the American (16.9%) and Pakistani end users (9.6%). It thus highlights the importance for end users to be familiar with the safe operation of ultrasound equipment.

**TABLE 2:** Opinions on safe ultrasound practice and knowledge of safety issues.

Opinions on safe ultrasound practice	Frequency (*N* = 121)	%
**How many scans should a woman undergo during a low-risk pregnancy?**		
1 scan	12	10
2 scans	36	30
3 scans	51	42
More than 3 scans	22	18
**There should be limitations regarding the number of ultrasound examinations that a ‘low-risk’ pregnant woman should have during pregnancy**		
Strongly disagree	24	20
Disagree	16	13
Neutral	12	10
Agree	28	23
Strongly agree	41	34
**Are there any adverse effects to the foetus during US examinations?**		
Yes	43	36
No	78	65
**Ultrasound is safe during the first trimester**		
Should only be used for medical reasons	28	23
Safe but should be used when medically indicated	55	46
Perfectly safe, no limitations	38	31
**Ultrasound is safe during the second trimester**		
Should only be used for medical reasons	15	12
Safe but should be used when medically indicated	55	46
Perfectly safe, no limitations	51	42
**Ultrasound is safe during the third trimester**		
Should only be used for medical reasons	15	12
Safe but should be used when medically indicated	55	46
Perfectly safe, no limitations	51	42
**Doppler ultrasound is safe during the first trimester**		
Should only be used for medical reasons	85	70
Safe but should be used when medically indicated	25	21
Perfectly safe, no limitations.	11	9
**Doppler ultrasound is safe during the second trimester**		
Should only be used for medical reasons	36	30
Safe but should be used when medically indicated	63	52
Perfectly safe, no limitations	22	18
**Doppler ultrasound is safe during the third trimester**		
Should only be used for medical reasons	29	24
Safe but should be used when medically indicated	60	50
Perfectly safe, no limitations	32	26
**Knowledge of safety issues**		
Familiar with TI	93	77
Familiar with MI	74	61
Familiar with TIC	38	31
Familiar with TIB	52	43
Knowledge of temperature with potential first trimester teratogenic effects	53	44
Familiarity with the term ‘ALARA’	60	50

*Source*: Sheiner, E., Shoham, V. & Abramowicz, J.S., 2007, ‘What do clinical users know regarding safety of ultrasound during pregnancy?’, *Journal of Ultrasound in Medicine* 26, 319–325. https://doi.org/10.7863/jum.2007.26.3.319

**TABLE 3:** Profession versus use of spectral/colour Doppler in the third trimester.

Professional	Doppler third trimester			Total
	Used only for medical reasons	Safe, but should be used mainly when medically indicated	Perfectly safe, no limitations	
Sonographer	12	9	2	23
General practitioner/Physician	2	5	2	9
Obstetrician and gynaecologist	14	44	28	86
Maternal-foetal medicine specialist	1	2	0	3

**Total**	**29**	**60**	**32**	**121**

*Source*: Sheiner, E., Shoham, V. & Abramowicz, J.S., 2007, ‘What do clinical users know regarding safety of ultrasound during pregnancy?’, *Journal of Ultrasound in Medicine* 26, 319–325. https://doi.org/10.7863/jum.2007.26.3.319

Pearson’s chi-square, *p* = 0.017.

In comparison with the results from previous studies, South African end users demonstrated better knowledge of TI (77%) and MI (61%) safety indices (Table 3). In a multicentre European study by Marsal (2005:212), only 22% of the respondents could define TI while only 11% could define MI. Similarly, Sheiner et al. (2007:321) published results from the American survey in which 32% and 22% were familiar with the TI and MI safety indices, respectively. In a more recent Pakistani survey (Akhtar et al. 2011:982), 34% of the respondents were familiar with the acronym TI and 32% with the acronym MI. Furthermore, in our study, a decline was noted in the response to the questions on the thermal index for cranial bone (TIC) (31%) and the thermal index for bone (TIB) (43%). However, our results were still better than the results of the European study completed by respondents from Sweden, Norway and Austria, which only achieved 8% and 3% (Marsal 2005:212), respectively, for the same questions, suggesting greater awareness of the safety indices among South African end users. It is possible that the results of our study would have been in keeping with the rest of the world if South African end users were similarly required to provide open-ended answers rather than selecting the appropriate response from a set of preselected answers.

When questioned on the safety of B-mode and Doppler ultrasound in the three trimesters, end users indicated caution in the first trimester in general and even more caution in the application of Doppler ultrasound as did their American counterparts. Around one-third of the respondents stated that B-mode ultrasound was perfectly safe in the first trimester and could be used without any limitations (Table 2), compared to 36% in the American study (Sheiner et al. 2007:322). In our study, the trend decreased when opinions were tested on the perception of Doppler safety, with only 9% of the end users stating that Doppler test was perfectly safe in the first trimester compared to 19% in the American study (Sheiner et al. 2007:322).

While there is a general consensus that embryonic tissue is most sensitive to heat damage during the first trimester (Bly & Van den Hof 2005:575) (Table 2), the heating effect in the third trimester is aggravated by the increased ossification in foetal bones, where the TI, in particular, can reach levels ≥ 1.5 when higher sound intensities are employed during middle cerebral artery (MCA) Doppler studies. It is of concern that only 42% of the respondents in our study stated that ultrasound was perfectly safe in the third trimester, while 26% shared the same sentiment about the use of Doppler ultrasound in the third trimester (Table 2). A general decline of favour towards Doppler ultrasound as opposed to B-mode imaging was observed in all three trimesters. A statistically significant difference (*p* = 0.017) was recorded between professional categories, with sonographers showing the greatest caution when using Doppler ultrasound in the third trimester (Table 4).

**TABLE 4:** Profession versus knowledge of the as low as reasonably achievable principle.

Professional	ALARA				Total
	As low as realistically attainable	As low as reasonably achievable	As low as realistically achievable	As low as reasonably attainable	
Sonographer	0	21	0	2	23
General practitioner/Physician	0	6	1	2	9
Obstetrician and gynaecologist	9	32	12	33	86
Maternal-foetal medicine specialist	0	1	0	2	3

**Total**	**9**	**60**	**13**	**39**	**121**

ALARA, as low as reasonably achievable.

Pearson’s chi-square, *p* = 0.003.

Although traditionally used in terms of ionising radiation, the application of the ALARA principle is now widely advocated in the prudent use of obstetric ultrasound (Miller 2008:159). It was disappointing that only half of therespondents in our study knew the correct meaning of the acronym. A significant statistical difference was noted (*p* = 0.03) between professional categories, with 91% of sonographers demonstrating familiarity with the term (Table 5). This can most likely be attributed to their knowledge gained from diagnostic radiography where the concept is pivotal in radiation safety. Furthermore, there was a positive association between the number of scans performed by end users and the correct identification of the term ALARA (*p* = 0.02) (Table 6).

**TABLE 5:** Number of scans performed daily versus knowledge of the as low as reasonably achievable principle.

Daily number of ultrasounds	ALARA				Total
	As low as realistically attainable	As low as reasonably achievable	As low as realistically achievable	As low as reasonably attainable	
0–2 scans	0	7	2	1	10
3–5 scans	3	9	5	7	24
6–10 scans	2	16	3	9	30
11–15 scans	4	10	1	20	35
More than 15 scans	0	18	2	2	22

**Total**	**9**	**60**	**13**	**39**	**121**

ALARA, as low as reasonably achievable.

Pearson’s chi-square, *p* = 0.02

**TABLE 6:** Experience in ultrasound versus opinion on limitation of scans in low-risk pregnancies.

Experience in ultrasound	Limitation in low-risk scans					Total
	Strongly disagree	Disagree	Neutral	Agree	Strongly agree	
Less than 1 year	0	0	1	0	1	2
1–2 years	1	2	0	2	4	9
3–5 years	2	2	1	9	10	24
6–10 years	5	0	3	7	14	29
11–15 years	7	4	0	8	2	21
16–20 years	5	5	4	0	7	21
More than 20 years	4	3	3	2	3	15

**Total**	**24**	**16**	**12**	**28**	**41**	**121**

*Source*: Sheiner, E., Shoham, V. & Abramowicz, J.S., 2007, ‘What do clinical users know regarding safety of ultrasound during pregnancy?’, *Journal of Ultrasound in Medicine* 26, 319–325. https://doi.org/10.7863/jum.2007.26.3.319

Pearson’s chi-square, *p* = 0.019

There has been ongoing debate over the past three decades on the benefits of routine ultrasound screening and the ideal number of ultrasound scans performed in a low-risk pregnancy (Alfiveric, Stampalja & Medley 2015). Although the routine use of ultrasound in low-risk pregnancies is now widely regarded as an integral part of antenatal care (Alfiveric et al. 2015), the expectation remains that it should be used only when medically indicated. In addition, the exposure should be controlled by using the lowest possible output settings and by limiting the scanning time and the number of scans (BMUS 2012; EFSUMB 2018).

Financial gain and parental expectations have been identified as some of the motivating factors behind frequent ultrasound scans (Helliker 2015). In our study, only 18% of the respondents advocated for more than three scans in a low-risk pregnancy. Similarly, the majority of Americans deemed a mean of 2.6 scans to be ideal in a low-risk pregnancy (Sheiner et al. 2007:322). Similar results were noted between the two study populations on the issue of limiting the frequency of ultrasound scans, with 57% South Africans agreeing and strongly agreeing with the limitation in the number of scans in a low-risk pregnancy and 50% of the Americans who expressed the same view. A statistically significant association (*p* = 0.019) was found between the years of experience and the number of routine scans deemed suitable during pregnancy (Table 6), with more experienced South African professionals being more cautious with the use of ultrasound.

## Conclusions, limitations and recommendations for future research

For the ODS to be effective, it is crucial that operators are knowledgeable about the possible bio-effects of diagnostic ultrasound (Marsal 2005:214). Although knowledge levels in our study were higher than that in previous surveys, it is discouraging to see that end users who are entrusted with controlling ultrasound exposure to the foetus still demonstrate poor knowledge of the basic aspects of ultrasound safety. Knowledge of TI and MI on its own is insufficient to monitor patient safety because awareness of all factors contributing to adverse biological effects and knowledge of how to minimise these effects and monitor the TI and MI are all essential elements of the ALARA principle (Bagley et al. 2011:253). This is especially of concern as the responsibility for the cautious use of ultrasound has been shifted to the operator, implying that operators have to balance the risk of making an inaccurate diagnosis against the potential biological risk from ultrasound exposure. It is thus imperative that the appropriate training of end users is reinforced and awareness is raised on every available platform. It is for this reason that ISUOG (Marsal 2005:213) recommends a compulsory session on ultrasound safety at all affiliated congresses and seminars.

**FIGURE 1:**
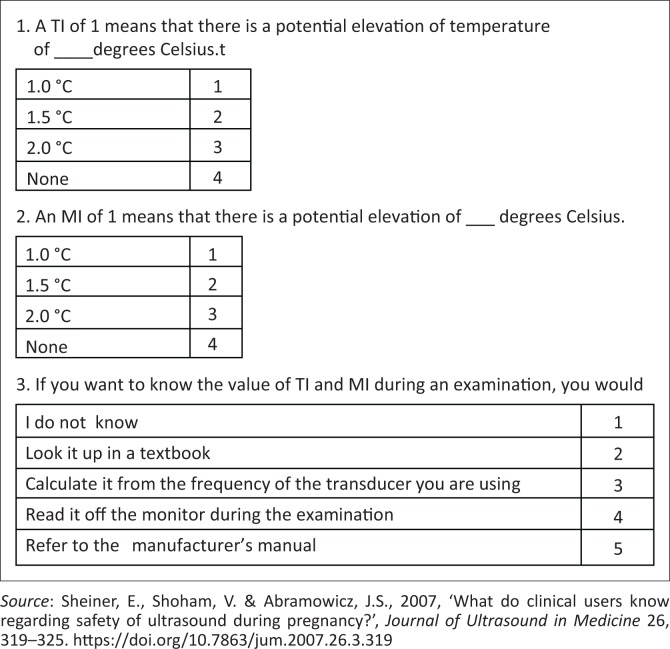
Data collection instrument.

Although the main aim of the study was achieved, the study design and research process were not without flaws or limitations. The data collection instrument was flawed by the omission of important knowledge questions because of a printing error; these are depicted in Figure 1.

However the results indicate that there would have been no significant difference in the outcome of the study. In retrospect, a question ascertaining whether formal ultrasound training was received at any point could have been included as per the study by Houston, Allsworth and Macones (2011:24).

A follow-up study, investigating the knowledge of application specialists who represent the manufacturers of ultrasound equipment, would be of value as vendors are required by the ODS to supply end users with sufficient knowledge about bio-effects when marketing ultrasound units. The results of such a study could provide a reflection of whether manufacturers are playing their role as mandated by the FDA and whether this, in addition to possible lack of training, could be the main cause of the poor knowledge of bio-effects in our own and previous studies. Future studies could additionally focus on professional categories to ascertain whether the desired learning outcomes are being met for each discipline.

With the demand for obstetric ultrasound examinations on the rise and in the absence of the assurance that ultrasound is as safe as generally believed, a concerted effort should be made to draw attention to this issue and address safety concerns that currently exists among end users of this very useful modality.

## Appendix 1: Questionnaire investigating the knowledge of health practitioners who use ultrasound equipment regarding the safety of ultrasound

**Example of how to complete this questionnaire:** Gender

1. If you are male, then answer as follows:
  **Table:**
  Male	1
  Female	2
  Age (complete in years)
2. If you are 10 years old, then answer as follows:
  **Table:**
  1	0

### SECTION A

#### General biographical information

1. Do you perform obstetric scans?
  **Table:**
  Yes	1
  No	2
  **If you answered ‘No’ to the above question, you are NOT required to complete the rest of the questionnaire. Thank you for your time.**
2. Gender
  **Table:**
  Male	1
  Female	2
3. Age (please choose the relevant category)
  **Table:**
  Younger than 20 years	1
  21–30 years	2
  31–40 years	3
  41–50 years	4
  Older than 59 years	5
4. Number of children
  **Table:**
  0	1
  1–2 children	2
  3–4 children	3
  5–6 children	4
  More than 6 children	5
5. Do you reside in South Africa?
  **Table:**
  Yes	1
  No	2
  If you answered no to the above question, please specify your country
  ________________________________________________________
6. If residing in South Africa, which province do you practise in?
  **Table:**
  Gauteng	1
  Mpumalanga	2
  KwaZulu-Natal	3
  Eastern Cape	4
  Western Cape	5
  Northern Cape	6
  Limpopo	7
  Free State	8
  North West	9
7. Please state your profession.
  **Table:**
  Sonographer	1
  Nurse (general)	2
  Midwife	3
  General practitioner/Physician	4
  Obstetrician and gynaecologist	5
  Materno-foetal medicine specialist	6
  Radiologist	7
  Other	8
  If other, please specify_________________________________
8. Years of experience in your profession?
  **Table:**
  Less than 1 year	1
  1–2 years	2
  3–5 years	3
  6–10 years	4
  11–15 years	5
  15–20 years	6
  More than 20 years	7
9. Years of experience in ultrasound.
  **Table:**
  Less than 1 year	1
  1–2 years	2
  3–5 years	3
  6–10 years	4
  11–15 years	5
  15–20 years	6
  More than 20 years	7
10. Place of work?
  **Table:**
  Government hospital	1
  Private hospital	2
  Other	3
  If other please specify ____________________________

### SECTION B

#### Participant’s opinion regarding scanning and referral practices

11. Did you or your partner have any obstetric scans during pregnancy?
  **Table:**
  Yes	1
  No	2
  If you answered yes to the above question, how many scans did you or your partner have?
  **Table:**
  1–2 scans	1
  3–4 scans	2
  4–5 scans	3
  5–6 scans	4
  More than 6 scans	5
12. Year of most recent pregnancy? For example 1999
  **Table:**
13. Average number of ultrasound examinations you perform per day?
  **Table:**
  0–2 scans	1
  3–5 scans	2
  6–10 scans	3
  11–15 scans	4
  More than 16 scans	5
14. According to safety guidelines how many scans should a woman undergo during a ‘low-risk’ pregnancy?
  **Table:**
  None	1
  1 scan	2
  2 scans	3
  3 scans	4
  More than 3 scans	5
15. There should be limitation regarding the number of ultrasound examinations that a ‘low-risk’ pregnant woman should have during her pregnancy?
  **Table:**
  Strongly disagree	1
  Disagree	2
  Somewhat disagree	3
  Somewhat agree	4
  Agree	5
  Strongly agree	6
16. Are there any adverse effects to the foetus during ultrasound examinations?
  **Table:**
  Yes	1
  No	2
  If yes please specify. ____________________________________________
17. Please answer questions 17–22 according to the key provided:
  **Table:**
  	Should be used only for medical reasons	Safe but should be used mainly when medically indicated	Perfectly safe, no limitations
  17. Ultrasound is safe during the first trimester	1	2	3
  18. Ultrasound is safe during the second trimester	1	2	3
  19. Ultrasound is safe during the third trimester	1	2	3
  20. Doppler ultrasound is safe during the first trimester	1	2	3
  21. Doppler ultrasound is safe during the second trimester	1	2	3
  22. Doppler ultrasound is safe during the third trimester	1	2	3

### SECTION C

#### Familiarly with ultrasound bio-effects, output display standards, safety indices and safety statements. *Questions in this section are in relation to obstetric ultrasound safety.

23. What does TI stand for?
  **Table:**
  Temperature increase	1
  Temperature index	2
  Thermal index	3
  Thermal insonation	4
24. What does MI stand for?
  **Table:**
  Mechanical increase	1
  Mechanical index	2
  Mechanical input	3
  Mechanical insonation	4
25. What does TIC stand for?
  **Table:**
  Temperature increase in cranium	1
  Thermal increase in cavity	2
  Thermal index in cranium	3
  Temperature index in cavity	4
26. What does TIB stand for?
  **Table:**
  Thermal increase in body	1
  Thermal increase in bone	2
  Temperature increase in body	3
  Thermal index in bone	4
27. How often do you perform Doppler studies during the first trimester of pregnancy?
  **Table:**
  Never	1
  Sometimes	2
  Always	3
  If you answered sometimes to always, please tick the relevant reason?
  **Table:**
  Hear heartbeat	1
  Ductus venosus assessment	2
  Tricuspid valve assessment	3
  Uterine artery assessment	4
28. It is acceptable to perform keepsake ultrasound (souvenir photos of the unborn child performed in a non-medical facility)
  **Table:**
  Totally unacceptable	1
  Unacceptable	2
  Slightly unacceptable	3
  Slightly acceptable	4
  Acceptable	5
  Perfectly acceptable	6
29. What elevation of temperature (in degrees Celsius) could potentially be teratogenic during the first trimester of pregnancy?
  **Table:**
  None	1
  0.5	2
  1	3
  1.5	4
30. What does the term ALARA stand for?
  **Table:**
  As low as realistically attainable	1
  As low as reasonably achievable	2
  As low as realistically achievable	3
  As low as reasonably attainable	4
